# Clinically used antirheumatic agent auranofin is a proteasomal deubiquitinase inhibitor and inhibits tumor growth

**DOI:** 10.18632/oncotarget.2113

**Published:** 2014-06-18

**Authors:** Ningning Liu, Xiaofen Li, Hongbiao Huang, Chong Zhao, Siyan Liao, Changshan Yang, Shouting Liu, Wenbin Song, Xiaoyu Lu, Xiaoying Lan, Xin Chen, Songgang Yi, Li Xu, Lili Jiang, Canguo Zhao, Xiaoxian Dong, Ping Zhou, Shujue Li, Shunqing Wang, Xianping Shi, Ping Q. Dou, Xuejun Wang, Jinbao Liu

**Affiliations:** ^1^ State Key Lab of Respiratory Disease, Protein Modification and Degradation Lab, Department of Pathophysiology, Guangzhou Medical University, Guangdong, China; ^2^ Guangzhou Research Institute of Cardiovascular Disease, the Second Affiliated Hospital, Guangzhou Medical University, Guangzhou, Guangdong, People's Republic of China; ^3^ Department of Hematology, The People's Hospital of Guangxi Autonomous Region, Nanning, Guangxi, People's Republic of China; ^4^ Guangzhou Key Laboratory of Urology, The First Affiliated Hospital of Guangzhou Medical University, Guangzhou, Guangdong, People's Republic of China; ^5^ The Molecular Therapeutics Program, Barbara Ann Karmanos Cancer Institute, and Departments of Oncology, Pharmacology and Pathology, School of Medicine, Wayne State University, Detroit, Michigan, USA; ^6^ Division of Basic Biomedical Sciences, Sanford School of Medicine of the University of South Dakota, Vermillion, South Dakota, USA

**Keywords:** cancer, deubiquitinase, proteasome, auranofin

## Abstract

Proteasomes are attractive emerging targets for anti-cancer therapies. Auranofin (Aur), a gold-containing compound clinically used to treat rheumatic arthritis, was recently approved by US Food and Drug Administration for Phase II clinical trial to treat cancer but its anti-cancer mechanism is poorly understood. Here we report that (i) Aur shows proteasome-inhibitory effect that is comparable to that of bortezomib/Velcade (Vel); (ii) different from bortezomib, Aur inhibits proteasome-associated deubiquitinases (DUBs) UCHL5 and USP14 rather than the 20S proteasome; (iii) inhibition of the proteasome-associated DUBs is required for Aur-induced cytotoxicity; and (iv) Aur selectively inhibits tumor growth *in vivo* and induces cytotoxicity in cancer cells from acute myeloid leukemia patients. This study provides important novel insight into understanding the proteasome-inhibiting property of metal-containing compounds. Although several DUB inhibitors were reported, this study uncovers the first drug already used in clinic that can inhibit proteasome-associated DUBs with promising anti-tumor effects.

## INTRODUCTION

The degradation by the ubiquitin-proteasome system (UPS) is a tightly regulated process responsible for maintenance of protein homeostasis in cells. The 26S proteasome consists of both 19S regulatory particles (RP) and the 20S core particle (CP). Increased proteasome activity has been reported in many different cancers, such as colon and prostate cancers and leukemia, suggesting that cancer cells may rely more heavily on the UPS than non-cancer cells. Targeting this pathway was validated as a strategy by the FDA (Food and Drug Administration) approval of bortezomib/Velcade (Vel) for the treatment of relapsed multiple myeloma and mantle cell lymphoma [1]. Therefore, both 20S proteasome peptidases and the 19S proteasome-associated deubiquitinases (DUBs) are becoming attractive targets of cancer therapy.

DUBs are proteases that cleave ubiquitin or ubiquitin-like proteins from ubiquitin pro-proteins or conjugates with target proteins. There are 98 putative ubiquitin-specific DUBs encoded by the human genome, which are classified to six different families based on sequence and structural features of their DUB active sites [2-4]. Five families belong to cysteine proteases: ubiquitin C-terminal hydrolase (UCH), ubiquitin specific protease (USP), ovarian tumor domain protease (OTU), Josephin domain protease (MJD) and monocyte chemotactic protein-induced protein family (MCPIP). The other family of DUBs belongs to the JAB1/MPN/Mov34 metalloenzyme (JAMM) domain family of Zn^2+^-dependent metalloproteases [4, 5]. Deubiquitination is implicated in many cellular processes, including cell cycle regulation [6], protein degradation [7], gene expression [8], and DNA repair [9, 10]. Mutations in several DUBs have also been linked to human diseases including cancer and neurological disorders [5, 11-13]. In humans, three DUBs are associated with the 19S RP. Two of them, UCHL5/Uch37 and USP14/Ubp6, are cysteine proteases of the UCH and USP families, respectively. The third DUB, RPN11/POH1, is a Zn^2+^-dependent protease of the JAMM family. RPN11 is a stoichiometric subunit of the lid of the 19S RP. The physiological roles of the 19S DUBs are not completely understood. It has been suggested that RPN11 performs ubiquitin chain amputation by cleaving the entire ubiquitin chain from the substrate in a process coupled to degradation [14, 15]. In contrast, the two cysteine-containing DUBs USP14 and UCHL5 trim ubiquitin chains from the distal end in a process antagonizing proteasomal degradation [16, 17]. It is generally believed that USP14 and UCHL5 provide a quality control function, ensuring short or non-degradable ubiquitinated substrates to be released from the proteasome [18]. Several DUBs have been found to be involved in cancer progression and therefore are emerging targets for anti-cancer therapies [19]. Of the three DUBs associated with the 19S RP, RPN11 is an obvious target for drug discovery due to its absolute requirement for cell survival [20]. The dependence of cell viability on RPN11 has been attributed to the DUB activity located in the JAMM motif of RPN11. RPN11 knockdown produces a similar phenotype to proteasome inhibition [16, 21]. In addition to RPN11, UCHL5 and USP14 are also associated with cell survival and cancer progession [22, 23].

We and others have reported that metal-containing compounds could induce cytotoxicity in human cancer cells *via* targeting the proteasome peptidases [24-26]. Several Zn, Cu compounds were toxic to cancer cells, associated with inhibition of cellular 26S proteasomes. Some of these metal compounds showed much less inhibitory effects against purified 20S proteasomes than against cellular 26S proteasomes [24, 25, 27]. It has been proposed that inhibition of DUBs in the 19S RP is possibly responsible for the anti-tumor effect of these metal complexes observed in cancer cells [24, 25, 27], but this hypothesis has not been tested.

Auranofin (Aur), a gold-containing compound, has been used clinically to treat rheumatic arthritis since 1985. It has also been reported that Aur has anti-cancer effects [28-30]. Aur was recently approved by FDA for Phase II clinical trial in cancer therapy (http://clinicaltrials.gov/ct2/show/NCT01419691). However, the mechanism underlying its anti-cancer effects remains poorly understood. Previous studies identified several potential molecular targets for the anti-inflammatory and anti-cancer activities of Aur [31-36]. One of the earlier studies suggested that Aur inhibits DNA synthesis, RNA synthesis, and protein synthesis, while later studies added several other targets including reactive oxygen species (ROS), mitochondrial thioredoxin reductase, glutathione-S-transferase, and cathepsin B. When we carefully analyzed the cytotoxic effect of Aur and its reported mechanisms, it became apparent to us that some of the characteristics induced by Aur are very consistent with the changes induced by proteasome inhibition; thus we propose that like copper compounds, Aur may target the proteasome.

Here we provide compelling evidence that Aur, a gold-containing compound, inhibits the proteasome *via* targeting proteasome-associated DUBs but not 20S proteasome peptidases, a mechanism distinct to the FDA approved proteasome inhibitor bortezomib, and that the inhibition of proteasome-associated DUBs is required for Aur-mediated cytotoxicity, unveiling a new fundamental mechanism for the anti-cancer effects of Aur.

## RESULTS

### Aur induces apoptosis in HepG2 and MCF-7 cells

**Figure 1:**
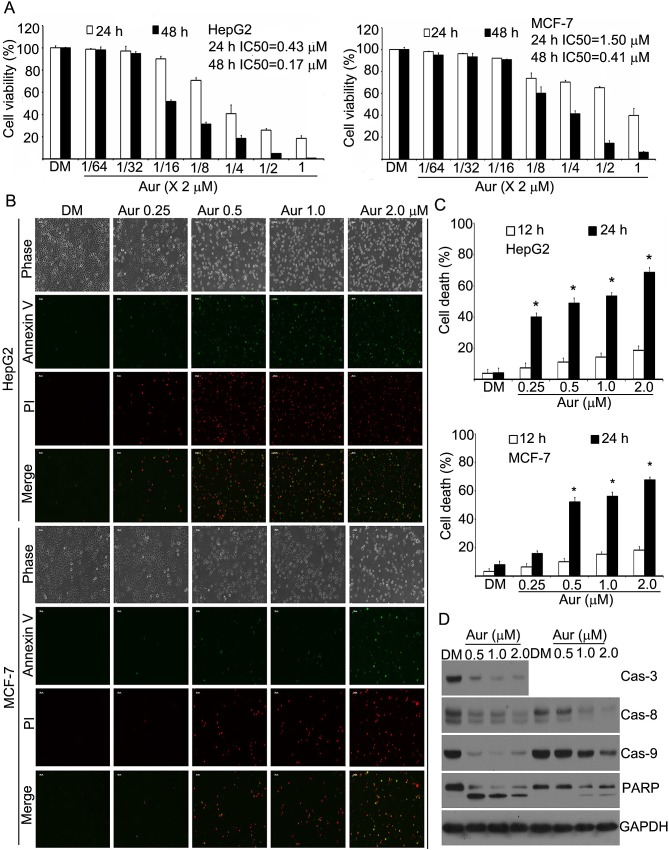
Auranofin (Aur) induces cell apoptosis in human HepG2 and MCF-7 cells (A) Cytotoxic effects of Aur on HepG2 and MCF-7 cells. HepG2 and MCF-7 cells were exposed to Aur in various concentrations for 24 or 48 h, and then were subjected to MTS assay. Data from three biological repeats are presented. Mean±SD (n=3). (B, C) Cell death induction by Aur in HepG2 and MCF-7. HepG2 and MCF-7 cells were treated with different doses of Aur for 12 or 24 h, then apoptotic cells were detected by Annexin V-FITC / Propidium iodide (PI) double staining, and the stained cells were either recorded using an inverted fluorescence microscope (Axio Obsever Z1, Zeiss, Germany) or detected by flow cytometry (FACScan, Becton-Dickinson). Representative images of the 24 h time point are shown in (B). Cell death data at 12 and 24 h are summarized in (C). Mean±SD (n=3). *P<0.05, compared with DMSO (DM) treatment. (D) PARP cleavage and caspase activation induced by Aur. HepG2 cells (left) and MCF-7 cells (right) were treated with Aur at the indicated doses for 18 h and then pro-caspases and PARP were detected by Western blot. GAPDH was used as a loading control.

To investigate the effect of Aur on the growth of human cancer cells, cultured HepG2 and MCF-7 cells were treated with Aur at various concentrations for 24 or 48 h and cell viability was measured with the MTS assay. As shown in Fig. 1A, Aur decreased the cell viability in a dose-dependent manner with the IC_50_ values of 0.43 (24 h) and 0.17 μM (48 h) in HepG2 cells and 1.5 (24 h) and 0.41 μM (48 h) in MCF-7 cells, respectively.

We next analyzed the capacity of Aur to induce cell death in these two cell lines. HepG2 and MCF-7 cells were exposed to Aur for either 12 or 24 h, followed by recording the Annexin V/PI (propidium iodide)-positive cells with fluorescence microscopy or flow cytometry. A dose-dependent cell death was observed (Figs. 1B and 1C). Consistently, the levels of the precursor forms of caspase-3, -8 and -9 were decreased after Aur treatment (MCF-7 cells do not express caspase 3), matching the pattern of PARP cleavage, which demonstrates that Aur triggers apoptosis *via* caspase activation (Fig. 1D).

### Aur inhibits the proteasome

**Figure 2:**
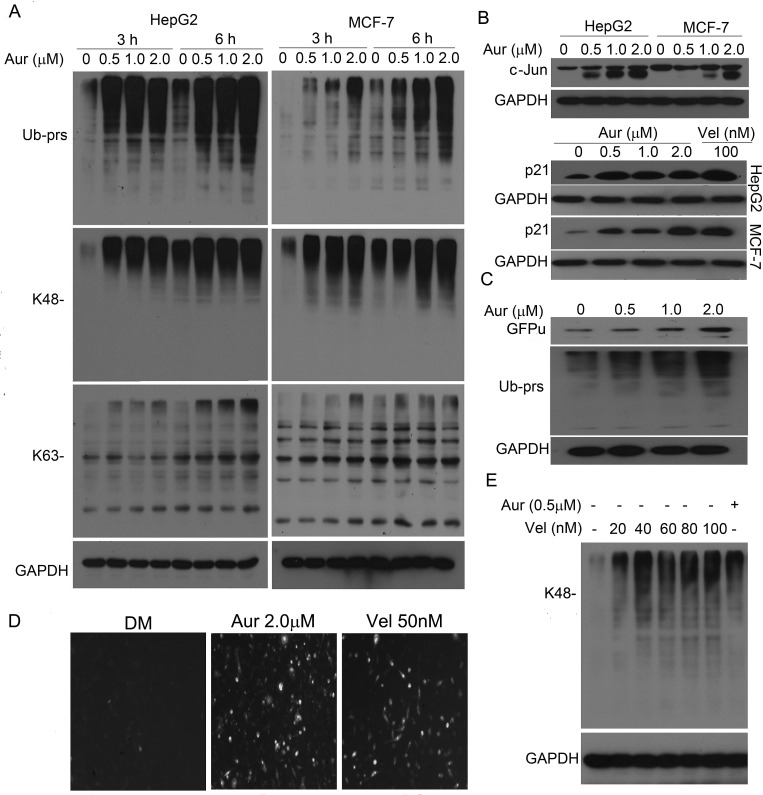
Aur inhibits the proteasome function (A) Accumulation of ubiquitinated proteins (Ub-prs). HepG2 and MCF-7 cells were exposed to Aur (0.5, 1.0, 2.0 μM) for 3 h and 6 h. Ub-prs were detected using antibodies against all Ub, K48-linked, or K63-linked polyubiquitin. GAPDH was used as a loading control. The western blot images were representatives from at least three independent experiments. (B) Accumulation of endogenous proteasome substrates. p21 and c-Jun proteins were detected after treatment with Aur (0.5, 1.0, 2.0 μM) or bortezomib/Velcade (Vel, 100 nM) for 9 h in both HepG2 and MCF-7 cells. (C, D) Accumulation of GFPu, a surrogate proteasome substrate. GFPu-HEK293 cells, a clonal HEK293 cell line stably transfected with GFPu (a surrogate UPS substrate created by carboxyl fusion of an enhanced green fluorescence protein with degron CL1), were treated with Aur (0.5, 1.0, 2.0 μM) for 6 h and then GFPu and Ub-prs were detected by western blot (C). Fluorescent GFPu images in the GFPu-HEK293 cells treated with Aur (2.0 μM) or Vel (50 nM) were shown in (D). (E) Comparison of the accumulation of K48-linked Ub-prs induced by Aur and Vel. K562 cells were treated with the indicated doses of Vel and Aur (0.5 μM) for 9 h and then K48-linked Ub-prs were detected by western blot analysis.

We and others have reported that gold (III)-containing compounds, like other metal (Cu, Zn) compounds, could directly inhibit 20S proteasome peptidase activities, but gold (I) compound was less effective [24-26]. We first determined the effect of Aur on endogenous proteasome substrate proteins in human HepG2 and MCF-7 cancer cells to assess its effect on the UPS. We found that Aur induced marked increases in total, K48- and K63-linked ubiquitinated proteins (Ub-prs, Fig. 2A) and in the protein levels of cyclin-dependent kinase inhibitor p21 and c-Jun proteins (Fig. 2B). In addition, Aur also accumulated a surrogate proteasome substrate (GFPu) and Ub-prs in a stable GFPu-HEK293 cell line (Figs. 2C and 2D). Aur at 2.0 μM and bortezomib (Vel) at 50 nM showed the similar level of GFPu accumulation (Fig. 2D). We further compared the efficacy of proteasome inhibition by Aur to that of Vel. We found that Ub-prs accumulation induced by therapeutic dose of Aur (0.5 μM) was similar to Vel at doses between 20 and 40 nM in K562 cells (Fig. 2E). These results indicate that the UPS inhibition by Vel can be achieved by a therapeutic dose of Aur.

### Aur inhibits 19S proteasome-associated DUBs but not 20S proteasome peptidases

To differentiate the proteasome target, we first detected 20S proteasome peptidase activities *in vitro* and in live HepG2 and MCF-7 cells by using the Promega peptidase assay kit. We found that Aur at a dose as high as 10 μM did not inhibit the activities of the 26S proteasome peptidases including chymotrypsin-like (CT-like), trypsin-like and caspase-like (Fig. S1A); unlike Vel, all the three proteolytic peptidases including CT-like, caspase-like and trypsin-like were not significantly affected by Aur treatment in live cells under used conditions (Fig. S1B and S1C). Next we tested its effect on proteasome DUB activities.

**Figure 3:**
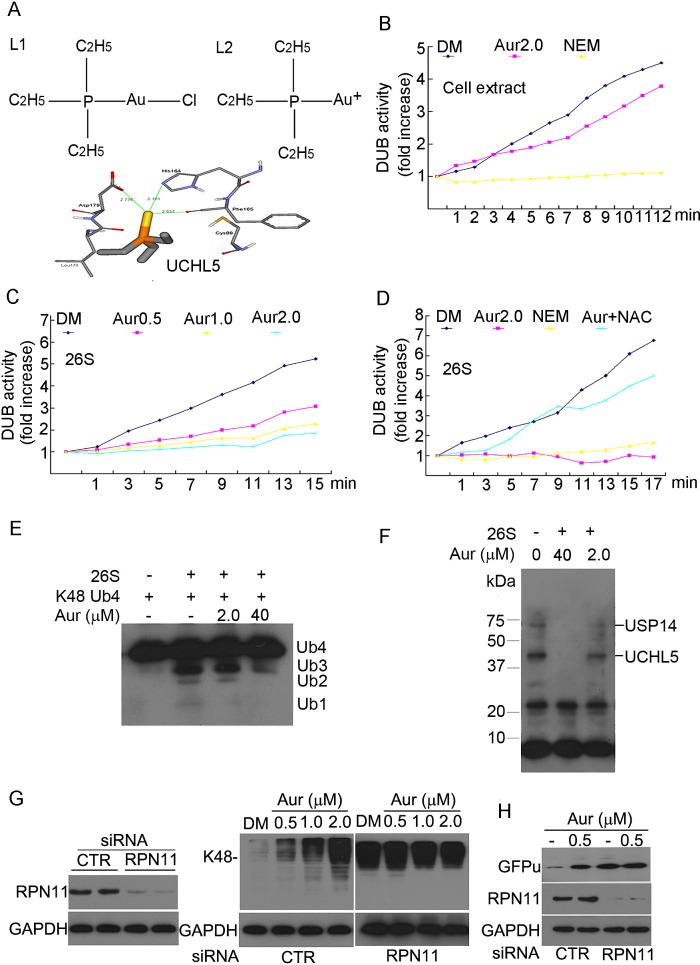
Aur inhibits the 19S proteasome DUB activity rather than 20S proteasome peptidases (A) Computational molecular docking of Au^+^ with UCHL5 of 19S proteasomes. The hydrolysed form of chloro (triethylphosphine) gold or Aur, (triethylphosphine) gold cation (L2, left), and its binding mode at the active site of UCHL5 were shown (right). (B) Effect of Aur on DUB activities in cell lysate. Cell lysate was treated with Aur (2μM) or NEM (N-ethylmaleimide, 2 mM), then the DUB activity at different times was recorded by using the Ub-AMC substrate. The experiment was repeated three times, yielding the similar results. (C) Inhibition of the DUB activity in 26S proteasomes. Purified 26S proteasomes were treated with increasing doses of Aur, then DUB activity was kinetically detected as in (B). (D) NAC rescues Aur-mediated DUB inhibition. Purified 26S proteasomes were treated with Aur (2 μM), Aur+NAC (100 μM), or NEM (2 mM) for 15 min, then DUB activity was detected. (E) Ubiquitin chain disassembly assay. K48-linked ubiquitin tetramers were disassembled by the 26S proteasomes after treatment with Aur (2.0, 40 μM). (F) Active-site–directed labeling of proteasomal DUBs. Purified 26S proteasomes were treated with Aur (2.0, 40 μM) for 10 min and then labeled with HA-UbVS and fractionated via SDS-PAGE. The covalently bound HA-UbVS was detected by western blot for the HA tag. (G) The effect of 26S proteasome disassembly by siRNA-mediated knockdown of RPN11 on Aur induced Ub-prs accumulation. HepG2 cells were transfected with specific siRNA against RPN11 for 48 h, and then treated with Aur (0.5, 1.0, 2.0 μM) for 6 h. Scrambled siRNA was used as control. K48-linked polyubiquitin and RPN11 protein was detected by western blot analyses. GAPDH was used as a loading control. (H) GFPu accumulation with RPN11 siRNA silencing and Aur treatment. GFPu-HEK293 cells were transfected with control siRNA or RPN11 siRNA for 48 h, and then treated with 0.5 μM Aur for 6 h. GFPu and RPN11 protein was detected by western blot analyses.

We first performed a computational study to predict the docking between Aur and the 19S-associated DUBs. It was found that compound L2 (an active metabolite of Aur, Fig. 3A, right) but not chloro triethylphosphine gold L1 (Fig. 3A, left), could bind to the active site of UCHL5 with relatively high CDOCKER Energy of -14.51 kcal/mol. The binding model (Fig. 3A, lower) shows that the side chains of His164, Phe165 and Asp179 can coordinate to Au^+^ with distances of 3.181 Å, 2.537 Å and 2.776Å, respectively. Moreover, two ethyl groups stretch towards hydrophobic side chains of Phe79 and Leu179. When compound L2 was docked to the active site of USP14, there were three ligand-poses produced (data not shown), suggesting that compound L2 could also inhibit USP14 activity but relatively less than UCHL5. The following experiments were performed to test this computational model.

First, total cytoplasmic DUB activities were detected by using Ub-AMC (ubiquitin 7-amido-4-methylcoumarin), a fluorogenic substrate for a wide range of DUBs, including UCHs and USPs. As shown in Fig. 3B, N-ethylmaleimide (NEM), an inhibitor of UCH and USP DUBs, completely inhibited, while Aur only slightly inhibited, the total cytoplasmic DUB activities. Secondly, we examined the effect of Aur on proteasome-associated DUBs by using Ub-AMC as a DUB substrate and purified 26S proteasomes as DUB donor. As shown in Fig. 3C, Aur (0.5, 1.0, 2.0 μM) inhibited proteasome DUB activity in a dose-dependent manner; Aur at a dose of 2.0 μM and NEM almost completely inhibited the 26S proteasome-associated DUB activities. Additionally, we used N-acetyl-cysteine (NAC), a thiol-containing compound to block the active site of Aur, and then tested its effect on DUB inhibition. Under physiological conditions, NAC could quickly bind with the Au+ atom of Aur forming a new compound AcS-Au (CH_3_)_3_ as detected by HPLC assay (Fig S2) and reported previously [38]. NAC recovered most Aur-mediated DUB inhibition (Fig. 3D). These results confirm the computational model that Aur targets both UCHL5 and USP14 of the 26S proteasome. The cleavage of tetraubiquitin chains (Ub4) mediated by 26S proteasome DUBs and a DUB active site-directed labelling assay were also performed to further confirm this effect. K48-linked Ub chains were cleaved in the presence of 26S proteasomes and this was partially blocked by Aur in a dose-dependent manner (Fig. 3E). To further decipher which proteasome-associated DUB is inhibited by Aur, we performed the active DUB labelling assay using HA-tagged ubiquitin-Vinyl Sulfone (HA-UbVS), a probe that can covalently bind to the active sites of the cysteine protease families of DUBs [17]. We found that the remaining active forms of both UCHL5 and USP14 (i.e., those can be covalently bound by HA-UbVS) were clearly reduced in the 26S proteasomes pre-treated with Aur at 2 μM and became completely undetectable in those pre-treated with 40 μM Aur (Fig. 3F), indicating that Aur inhibits both UCHL5 and USP14. Lastly, we employed the gene knockdown approach to disassemble 19S RP to test the necessity of 19S RP-associated DUBs as the target of Aur proteasome inhibition. It has been reported that RPN11 knockdown could disassemble the 19S RP of the 26S proteasome [39], which has also been confirmed in our study (data not shown) by glycerol gradient ultracentrifugation. In the current study, HepG2 or GFPu-HEK293 cells were transfected with RPN11-specific siRNA for 48 h and the effects of Aur on Ub-prs or GFPu accumulation were detected, respectively. As shown in Fig. 3G (left), RPN11 protein was effectively down-regulated with transfection of RPN11 siRNA. RPN11 knockdown itself highly induced Ub-prs accumulation which could not be further increased by Aur in HepG2 cancer cells (Fig. 3G, right). Similarly, Aur could not further increase GFPu accumulation mediated by RPN11 silencing in GFPu-HEK293 cells (Fig. 3H), further confirming that Aur inhibits the 26S proteasome via targeting UCHL5 and USP14.

### Proteasome inhibition is required for Aur-induced cytotoxicity

**Figure 4:**
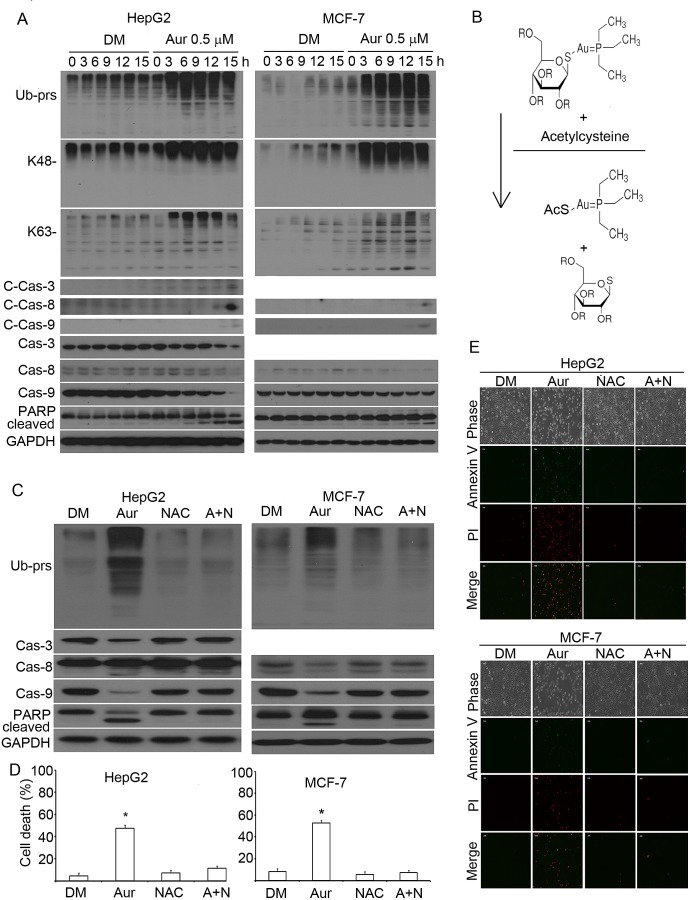
Proteasome inhibition is required for Aur to induce apoptosis (A) The time course of proteasome inhibition, caspase activation and apoptosis induction by Aur treament. HepG2 and MCF-7 cancer cells were treated with Aur (0.5 μM), then the cells were collected at the indicated time points for western blot analyses for ubiquitinated proteins including total ubiquitin conjugates, K48- and K63-linked polyubiquitins, as well as apoptosis-related proteins (caspases and PARP) in the whole cell lysate. C-Cas: cleaved caspases. GAPDH was used as a loading control. (B) An illustration of the binding of Aur with N-acetyl-L-cysteine (NAC) to inactivate Aur. In phosphate buffered saline (PBS), NAC binds with Aur, forming a new product. (C) NAC completely reversed Aur-induced proteasome inhibition and apoptosis. HepG2 and MCF-7 cells were treated with Aur, NAC, or Aur+NAC (A+N) for 18 h. Western blot analyses for the indicated proteins were performed. (D, E) NAC completely blocked Aur from inducing apoptosis. HepG2 and MCF-7 cells were treated as in (C) for 24 h, apoptotic cells were detected with Annexin V-PI staining followed by either flow cytometry (Mean±SD, n=3) or fluorescence microscopy. Flow cytometry data were summarized in (D), *P<0.05, *versus* Aur-treated alone. The phase contrast and fluorescent images were shown in (E). Red stain indicates PI-positive; green stain indicates Annexin V-positive. Scale bar=50 μm.

**Figure 5:**
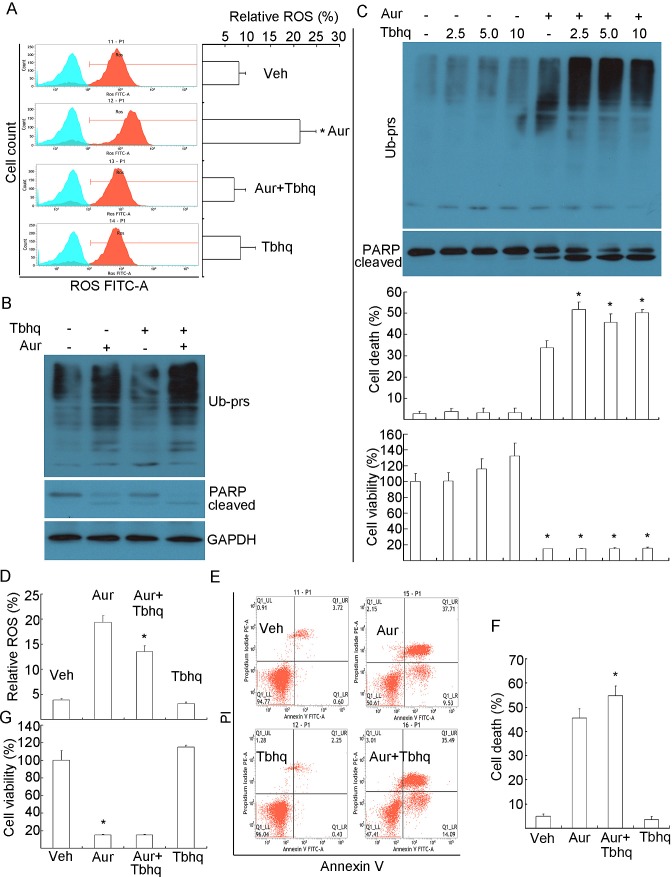
Phenol-containing antioxidant Tertiary butylhydroquinone (Tbhq) could scavenge Aur-induced ROS generation but could not rescue Aur-induced proteasome inhibition and apoptosis. (A, B) HepG2 cells were treated with Aur (0.5 μM), Tbhq (20 μM) or their combination for 12 h. ROS was detected by flow cytometry. Relative level of ROS was shown. Mean±SD (n=3). *P<0.05, compared with other treatments. (C) HepG2 cells were treated with increasing doses of Tbhq in the absence or presence of Aur (0.5 μM) for 24 h. Ubiquitinated proteins and PARP were detected by western blot analyses (upper). Cell death was detected by Annexin V/PI staining with flow cytometry. Mean±SD (n=3). *P<0.05, compared with Aur treatment alone. Cell viability was detected by MTS assay. Mean±SD (n=3). *P<0.05, compared with each treatment alone. (D) MCF-7 cells were treated with Aur (0.5 μM), Tbhq (5 μM) or their combination for 12 h. ROS was detected and shown as in (A). Mean±SD (n=3). *P<0.05, compared with Aur treatment alone, (E, F, G) MCF-7 cells were treated as in (D) for 24 h. Cell death and cell viability were detected as in (B). Cell death images and summary were shown in (E, F), and cell viability was shown in (G). Mean±SD (n=3). *P<0.05, compared with Aur control for cell death; compared with vehicle control for cell viability.

In these experiments, we first analysed the dynamic changes of proteasome inhibition and apoptosis induced by Aur. HepG2 and MCF-7 cells were treated with 0.5 μM Aur and then Ub-prs, caspases and PARP cleavage were detected at 3, 6, 9, 12, and 15 h time points. We found that Ub-prs accumulation (by proteasome inhibition) was induced at the early time point, followed by caspase activation and PARP cleavage (Fig. 4A), indicating apoptosis occurs after proteasome inhibition. Next, we used NAC (N-acetyl-cysteine) to block the active site of Aur and then tested the effect on Aur-induced proteasome inhibition and cell death. Similar to the rescuing effect of NAC on Aur-mediated DUB inhibition, NAC, by changing the chemical structure of Aur, completely reversed Aur-induced Ub-prs accumulation (Fig. 4B); and as expected, caspase activation and PARP cleavage (indicators of apoptosis) were accordingly abolished in HepG2 and MCF-7 cells (Fig. 4C). The effect on apoptosis was also confirmed using Annexin V/propidium (PI) staining followed by flow cytometry (Fig. 4D) or inverted fluorescent microscopy (Fig. 4E). Aur could also increase ROS (reactive oxygen species) production which could be blocked by NAC (Fig. S3) as previously reported [40]. To differentiate the effects of Aur-mediated DUB inhibition and ROS generation on cell apoptosis, we used a phenol-containing antioxidant, Tertiary butylhydroquinone (Tbhq), to compare the effects of Aur with NAC since, theoretically, Tbhq could not bind with the active atom site of Aur. As found, Tbhq (at 20 μM) could completely scavenge Aur-medaited ROS generation (Fig. 5A) but could not block Aur-mediated proteasome inhibition and PARP cleavage (apoptosis indicator) in HepG2 cells (Fig. 5B). Similar to 20 μM, relatively low doses of Tbhq could not block Aur-induced proteasome inhibition and cytotoxicity (Fig. 5C). In MCF-7 cells, 5 μM Tbhq could significantly decrease Aur-mediated ROS generation (Fig. 5D) but could not block Aur-mediated proteasome inhibition and cytotoxicity either (Fig. 5E-5G). These results further demonstrate that inhibition of DUB but not ROS is required for Aur-mediated cell death.

### Aur interferes with multiple proteasome-related signal pathways

**Figure 6:**
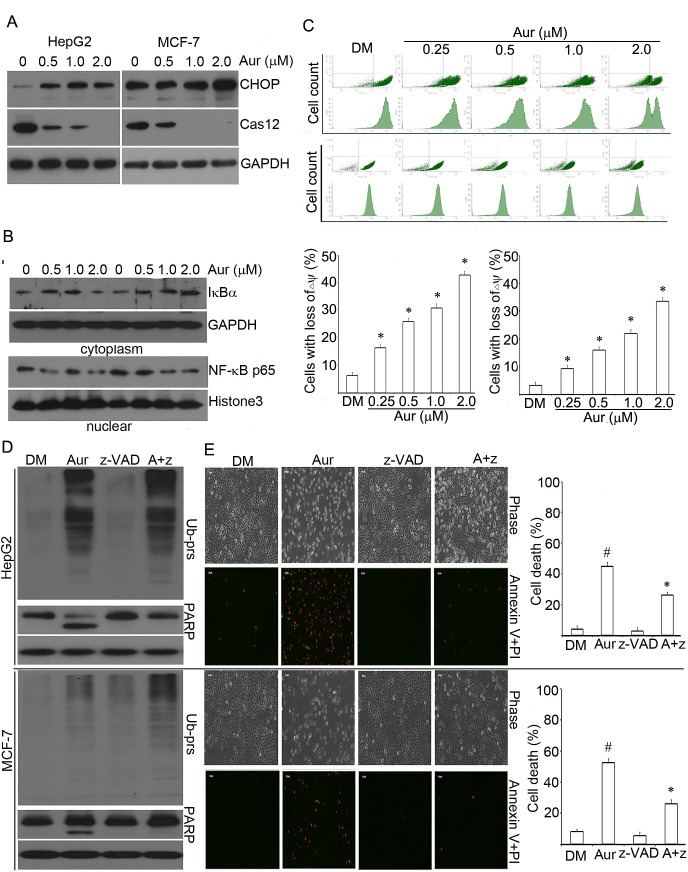
Aur interferes with multiple apoptosis-related signal pathways in cancer cells (A) CHOP and caspase 12 (Cas-12) protein expression. HepG2 and MCF-7 cells were exposed to Aur (0.5, 1.0, 2.0 μM) for 18 h. Western blot was performed for the detection of the ER stress-related proteins CHOP and Cas-12. (B) Changes in cytoplasmic IκBα and nuclear NF-κB p65 protein levels. HepG2 and MCF-7 cells were treated with Aur (0.5, 1.0, 2.0 μM) for 12 h. Cytoplasmic and nuclear proteins were extracted for western blot analyses for IκBα and NF-κB p65, respectively. GAPDH and histone 3 were used as cytoplasmic and nuclear protein loading control, respectively. (C) Mitochondrial membrane potential (ΔΨm) depolarization. As treated in (B), loss of ΔΨm was detected by flow cytometry. Representative flow images were shown (upper) and the quantitative data were summarized (lower). Mean±SD (n=3). *P<0.05, *versus* control. (D) HepG2 and MCF-7 cells were co-treated with Aur (0.5 μM) and z-VAD-FMK (50μM) for 18 h. Ub-prs and PARP proteins were assessed by western blotting. GAPDH was used as a loading control. (E) HepG2 and MCF-7 were treated as in (D) for 24 h, then apoptotic cells were detected with PI/annexin V staining, followed by either imaging under an inverted fluorescent microscope or detecting by flow cytometry. Representative phase contrast and fluorescent images were shown in (E, left). Red indicates PI-positive; green indicates annexin V-positive. Scale bar=50 μm. Cell death data by flow cytometry were shown in (E, right). Mean±SD (n = 3). #*P*<0.05, *versus* DM control; *P<0.05, *versus* Aur treatment alone.

Here we further investigated the effects of Aur on proteasome inhibition-related signal pathways. Several pathways, like ER (endoplasmic reticulum) stress and NF-κB inactivation, are involved in proteasome inhibition-induced cell death. We found that Aur treatment increased CHOP expression and induced caspase 12 activation in a dose-dependent fashion (Fig. 6A), indicating a sustained activation of the unfolded protein response (UPR). Aur treatment accumulated IκBα in the cytoplasm, thereby inhibiting the translocation of NF-κB from the cytoplasm to the nucleus (Fig. 6B). Mitochondrial membrane potential was also diminished in a dose-dependent manner by Aur treatment (Fig. 6C). These changes are consistent with the effects of proteasome inhibition observed in most previous reports [41]. Finally, we confirmed that Aur-induced cell death but not Ub-prs accumulation could be completely rescued by z-VAD, a pan-caspase inhibitor, as detected by PARP cleavage (Fig. 6D) and Annexin V/PI staining in both HepG2 and MCF-7 cells (Fig. 6E), indicating that like other classic proteasome inhibitors, Aur induces apoptosis mainly *via* caspase activation.

### Aur accumulates proteasome substrates and selectively inhibits tumor growth *in vivo*

**Figure 7:**
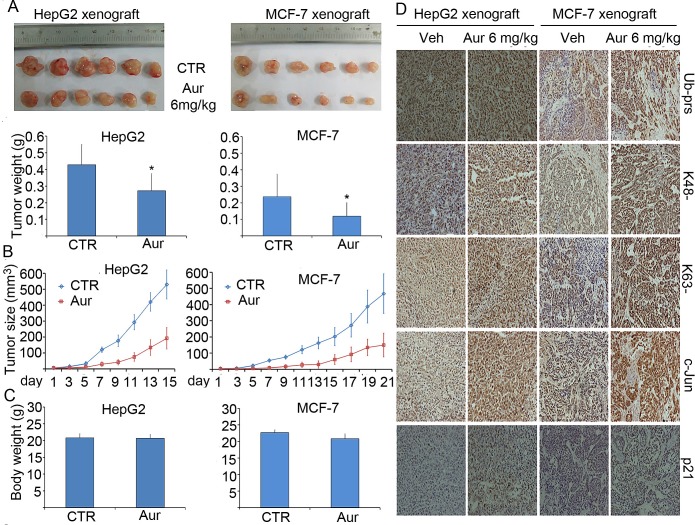
Aur inhibits tumor growth and the proteasome of tumor xenografts in mice without affecting mouse body weight BALB/c nude mice bearing HepG2 and MCF-7 tumors were treated with vehicle or Aur (6 mg/kg/day, i.p.) for 15 and 21 days, respectively. Tumor size was recorded every other day. Tumor images and tumor weight (A), tumor size (B) and body weight (C) and were shown. *P<0.05, compared with the control. (D) Representative micrographs of immunohistochemistry staining for total (Ub-prs), K48-linked (K48-), or K63-linked (K63-) ubiquitinated proteins and the indicated proteasome substrate proteins (c-Jun and p21) in nude mouse tumor tissues. All the immunostaining was repeated in three mouse tumor tissues and the images shown were collected at a magnification of 200×.

We next evaluated the effect of Aur *in vivo* using nude mouse xenograft models. We found that the tumor size curve and tumor growth curve were significantly different between Aur-treated- and vehicle-treated group in these models (Figs. 7A and 7B) and the weights of tumors were significantly reduced in Aur treatment group compared to the control (Figs. 7A and 7B) while body weight remained relatively stable in each group (Fig. 7C). The immunostaining results showed that the representative proteasome substrates including Ub-prs, p21, and c-Jun proteins were all significantly increased (Fig. 7D) in the Aur-treated tumors. Similar to the effect of Aur on cell lysate DUB activity, Aur did not significantly affect the total DUB activities in the tumor tissues (data not shown). These results are consistent with the effects of Aur observed in HepG2 and MCF-7 cells. Together, the results show that Aur selectively inhibits tumor growth and proteasome function *in vivo*.

### Aur induces cytotoxicity and proteasome malfunction in cancer cells from acute myeloid leukemia (AML) patients

**Figure 8:**
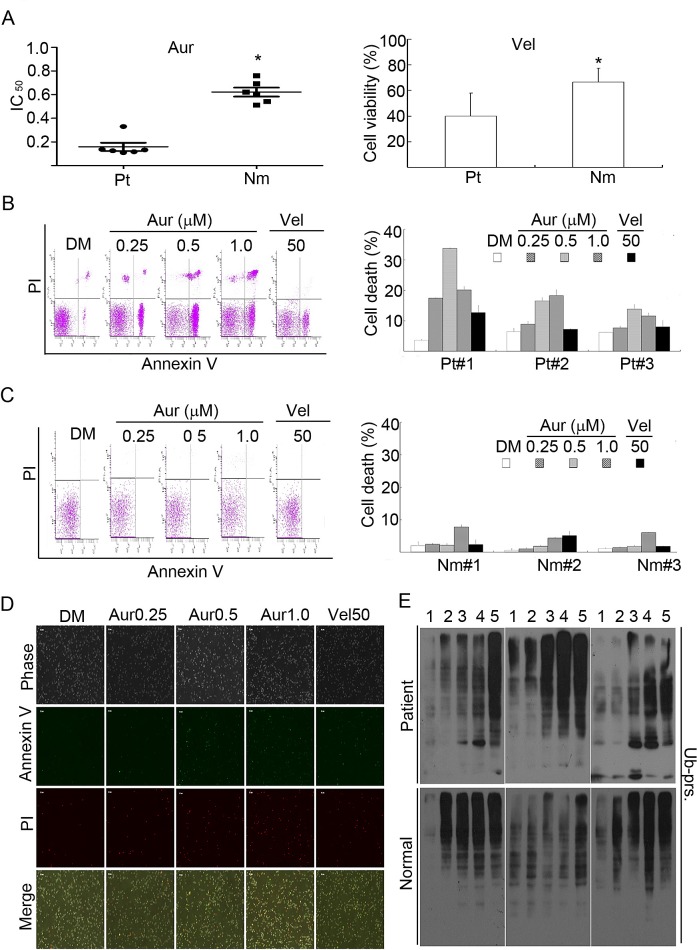
Aur inhibits the proteasome and specifically induces cytotoxicity in cancer cells from acute myeloid leukemia (AML) patients (A) Cancer cells from 6 AML patients (Pt) and peripheral blood mononuclear cells from 6 healthy volunteers (Nm) were treated with Aur at the indicated doses or with Vel (50 nM) for 24 h and the cell viability was detected by the MTS assay. The scatter plot of the IC_50_ values in each group was shown (A, left). *P<0.05, *versus* patients. Cell viability with Vel treatment in each group was shown (A, right). Mean±SD (n=3). *P<0.05, *versus* AML patients. (B, C) Cancer cells from 3 AML patients or the peripheral mononuclear cells from 3 normal human individuals were incubated with Aur at the indicated doses or with Vel (50 nM) for 12 h. Cell death was analyzed by flow cytometry. The typical images from flow cytometry were shown in (B, C, left) and cell death were summarized in (B, C, right). Mean±SD (n=3). (D) As treated in (B, C), cancer cells from AML patients were treated with Aur or Vel for 15 h, then cells were stained with Annexin V/PI and imaged under a fluorescent microscope. The phase contrast and fluorescent images were taken and merged. Scale bar=50 μm. (E) AML cancer cells and human peripheral mononuclear cells were treated with Aur or Vel for 6 h followed by detecting ubiquitinated proteins with western blot analyses. Western blot images of cells from 3 individuals of each group are shown. At the top of the panel, 1, 2, 3, 4, and 5 denote control, Aur (0.25, 0.5, 1.0 μM), and Vel (50 nM), respectively.

We next evaluated the *ex vivo* antineoplastic effect of Aur on bone marrow cells obtained from 6 patients with AML. Peripheral blood mononuclear cells from 6 healthy volunteers were used as controls. As shown in Fig. 8A (left), Aur decreased cell viability of primary monocytes from AML patients with IC_50_ values around 0.110-0.330 μM (average: 0.159 μM) while in normal controls its IC_50_ values were 0.513-0.761 μM (average: 0.622μM), similar to the effect of Vel (Fig. 8A, right). Aur treatment for 12 h at doses ranging from 0.25 to 1.0 μM resulted in significant apoptosis in the monocytes from AML patients as detected with Annexin V/PI staining followed by flow cytometry (Figs. 8B) or by fluorescence microscopy (Fig. 8D); however, similar treatment only caused minimal cell death in monocytes from healthy volunteers (Fig. 8C). Treatment with Aur significantly increased the level of Ub-prs in both cancer cells from AML patients and mononuclear cells from normal controls (Fig. 8E). These results demonstrate the *ex vitro* inhibitory effect of Aur on proteasome function and selective killing on AML cancer cells.

## DISCUSSION

DUBs especially proteasomal DUBs are emerging as attractive drug targets for cancer therapies. Although inhibitors of proteasomal DUBs were recently reported and shown experimentally to exhibit anticancer effects [42], their suitability for clinical use remains unknown. In the present study, we have discovered a novel property of Aur, which is that it inhibits proteasomal protein degradation by targeting primarily proteasomal DUBs. Moreover, we have further demonstrated that the anti-cancer effect of Aur depends on its DUB-inhibiting property. Hence, this study unveils the first DUB inhibitor that is already in clinical use to treat human disease.

The current study demonstrates that Aur inhibits the proteasome. This was confirmed by detecting both endogenous and exogenous proteasome substrate accumulation *in vitro*, *in vivo* and *ex vivo*, and this has also been verified by 19S RP disassembly. Proteasome inhibition induced by therapeutic doses of Aur is comparable to Vel by observing both Ub-prs and GFPu accumulation, like in some of the AML cells, therapeutic doses of Aur even more strongly inhibited proteasome inhibition than Vel. Aur inhibits the proteasome function, with mechanisms distinct to FDA approved proteasome inhibitor Vel. Unlike 20S proteasome inhibitors such as Vel, Aur did not inhibit the activities of chymotrypsin-like, trypsin-like and caspase-like activities of 20S proteasomes under the used experimental conditions.

We here show that Aur mainly targets proteasome-associated UCHL5 and USP14. Aur only slightly inhibits total cytoplasmic DUB activities but almost completely inhibits 26S proteasome UCHL5 and USP14 activities, similar to the effect of b-AP15, a confirmed UCHL5 and USP14 DUB inhibitor [17]. This is also confirmed by both K48-linked polyubiquitin disassembly, DUB active site-directed labeling assay, and 19S proteasome disassembly. It has been reported that caspase activation could inhibit proteasome function *via* cleaving 19S proteasome subunits [43]. In this study, we confirmed that Aur-mediated DUB inhibition is independent of caspase activation because pan-caspase inhibition prevented Aur from inducing apoptosis but did not stop Aur from accumulating Ub-Prs in cultured cells. It is known that Aur could induce intracellular ROS generation and inhibit thioredoxin reductase activity [30, 35], but H_2_O_2_ at a dose as high as 100 μM could not induce dramatic Ub-prs and GFPu accumulation like Aur (data not shown), implying that Aur-mediated proteasome inhibition is not associated with ROS generation. A most recent report found that ROS could directly inhibit only a small subsets of the cysteine-containing DUBs (like UCHL1, USP2) *via* thiol oxidation but not the metalloprotease like AMSH [44], further supporting that ROS could not exert an important contribution to Aur-induced DUB inhibition. However, whether Aur targets RPN11 remains unclear. Since there is no commercially available RPN11-specific substrate for its activity assay, the effect of Aur on RPN11 DUB activity could not be directly detected. Even though RPN11 knockdown could mostly blocked Aur-mediated DUB inhibition and protein degradation inhibition (Fig. 3G and 3H), 26S proteasome disassembly mediated by RPN11 knockdown is possibly the major reason. Based on the Ub chain cleavage data, 2 μM Aur could only partially inhibit the Ub chain cleavage, not as potent as in the cells. This is likely because the Ub chain cleavage relies on the existence of both UCHL5/USP14 and RPN11 in this *in vitro* assay since 26S proteasome consists of three DUBs and RPN11 could also cleave UB chain *in vitro* as reported previously [17]. We also found that high dose of Aur (40 μM) could completely block UbVS's binding with UCHL5 and USP14 but Aur at this dose could not completely stop proteasome-mediated Ub chain cleavage, indicating that RPN11 might not be a target of Aur, which however needs to be further investigated in the future. In this study, Aur induced the accumulation of both K48- and K63-liked polyubiquitinated proteins *in vitro* and *in vivo*. It is generally believed that cellular proteins conjugated to K48-linked Ub chains are targeted to the proteasome for degradation, while proteins conjugated to K63-linked Ub chains may be directed to lysosomes [45, 46]. To clarify this issue, a recent report shows that purified 26S proteasomes bind and degrade K48- and K63-ubiquitinated substrates similarly but in mammalian cells, soluble factors, such as ESCRT0, selectively bind to K63 chains, thereby inhibiting or preventing the association of K63 chains with the proteasome [47]. However, this was recently challenged by another report [48], suggesting that the regulation of K48- and K63-linked polyubiquitin remains elusive. It has been reported that Aur could inhibit lysosome protease cathepsin B, but the effective dose is much higher than the dose used in the present study [36]. This suggests that the accumulation of K63-linked poly ubiquitinated proteins by Aur is unlikely due to lysosome inhibition. Accumulation of K48-linked polyubiquitinated proteins is also an important indicator of 20S proteasome inhibition but this has been excluded by the inability of Aur to inhibit the 20S proteasome (Fig. S1). Taken together, these results indicate that Aur-mediated DUB inhibition induces accumulation of both K48- and K63-linked polyubiquitin *in vitro* and *in vivo*, with distinct effects to 20S proteasome inhibitors [49, 50].

Aur-induced proteasome inhibition is required for its cytotoxicity. It is well known that proteasome inhibition induces apoptosis. In Aur-treated cells, proteasome inhibition precedes apoptosis (Fig. 4a). Several laboratories have reported that the metabolic pathway of Aur most likely involves Au-S bond cleavage and thiol-containing compounds like GSH could replace glucopyranose and directly bind with Au atom to form a GS-Au-P-(CH_3_)_3_ compound [38]. This has also been verified in our study. Binding the active site of Aur with NAC not only prevents Aur from inhibiting DUB and the proteasome but also blocks Aur induction of apoptosis. Several mechanisms have been proposed to explain how proteasome-inhibition induces cell death. The induction of ER stress, loss of mitochondrial membrane potential, and suppression of NF-κB nuclear translocation by Aur are all consistent with a proteasome inhibition scenario, further supporting the notion that Aur induces cytotoxicity through inhibiting the proteasome. In spite of the numerous studies that appeared in the literature, the biological mechanisms of action of auranofin are still controversial. The most important mechanisms of the previous studies support that Aur targets thioredoxin reductase, thus inducing the generation of reactive oxygen species (ROS) and cell apoptosis [33-35]. Nevertheless some previous studies did not support this notion. Only Au (III) but not Aur could induce ROS generation [51]; The knockdown of thioredoxin reductase-1 (TrxR1), the well-known target of Aur, was not sufficient to oxidize thioredoxin-1 (Trx1), suggesting that Trx1-independent pathways should be considered when evaluating pharmacological and toxicological mechanisms involving TrxR1 inhibition [52]. In this current study, we did find that Aur could induce ROS generation in these cell lines but we confirm that ROS do not play important role in Aur-induced proteasome inhibition and cell apoptosis. This has been confirmed by (i) thepapeutic doses of Aur could dose-dependently inhibit proteasome DUB inhibition and apoptosis, which could be completely reversed by a classical ROS inhibitor NAC both *in vitro* and *in situ*. Two possibilities exist regarding the effect of NAC. On one hand, NAC scavenges ROS, on the other hand, thiol-containing antioxidant NAC blocks Aur's effect by binding with the active site of Aur. (ii) To differentiate the effects of ROS generation and DUB inhibition mediated by Aur, we used a phenol-containing antioxidant, Tbhq, to compare the effects of Aur with NAC since Tbhq theoretically could not bind with the active gold site of Aur. These two kinds of antioxidants could efficiently scavenge ROS. But these two different antioxidants have completely different effects on Aur-mediated proteasome inhibition and cell apoptosis. Thiol-containing antioxidant NAC could rescue Aur-mediated proteasome inhibition and cell apoptosis, while phenol-containing antioxidant Tbhq could scavenge ROS but could not rescue or even enhanced Aur-mediated proteasome inhibition and cell death. Tbhq itself did not dramatically affect cell viability. It is interesting to find that the combination of Tbhq and Aur showed enhanced proteasome inhibition and cell apoptosis, warranting further investigation in the future. These results further confirm that Aur-mediated apoptosis is associated with proteasome inhibition rather than ROS generation.

Cancer cells are more sensitive to proteasome inhibition. Here we also show that Aur inhibited tumor growth in human xenografts *in vivo* with minimal discernible toxicity, and Aur selectively induced cytotoxicity in primary cancer cells from AML patients. Aur treatment led to Ub-prs accumulation in normal mononuclear cells similarly to the cancer cells but the treatment induced much less cell death in the normal cells than in cancer cells. Previous reports that ATP bidirectionally regulates UPS is a possible explanation for this effect [53, 54].

Although several DUB inhibitors have been reported recently [17, 55, 56], a clinical DUB inhibitor has not been reported. During the screening for novel proteasome inhibitors, we discover that gold (I) compound Aur targets proteasome-specific DUBs and selectively induces cytotoxicity to cancer cells, while other metal-containing compounds such as copper complexes inhibit not only proteasome-associated DUBs but also non-proteasomal DUBs and 20S proteasome peptidases, thus inducing cytotoxicity to cancer cells not as selectively as Aur [57]. Hence, this study offers additional support to Gold (I) -containing compound Aur as a promising cancer drug candidate in cancer therapy. More importantly, this study provides new insight into the understanding on the relationship between metal-containing compounds and the UPS by demonstrating the DUB inhibition property of Aur and the necessity of the DUB inhibition in Aur-induced cytotoxicity and anti-tumor effects. To our best knowledge, Aur represents the first proteasome-specific DUB inhibitor that is in clinical use.

Studies into the molecular mechanisms of cancer have revealed that, with a few exceptions, the disease lacks a specific drug target. Therefore, new anticancer drugs not only take many years and much money to develop but also might not outperform existing drugs. Based on this paradigm, Blagosklonny has proposed a business model: to develop existing drugs for a novel use-the protection of normal cells. The drug discovery can be complemented by novel use of existing agents and even ‘failed’ drugs [58]. Certain drugs used for hypertension, atherosclerosis, diabetes, inflammation and immunosupression can protect against cancer. These drugs include rapamycin and other rapalogs, metformin, beta-blockers, angiotensin-blockers and aspirin [59, 60, 61, 62]. Aur has been used clinically to treat rheumatic arthritis for many years and it has also been recently approved by FDA for Phase II clinical trial in cancer therapy. In this current study, we have identified Aur as a potent proteasome deubiquitinase inhibitor and Aur-induced proteasome inhibition should be of great importance in the future clinical trials.

## METHODS

### Materials

Aur was purchased from Enzo Life Sciences International, Inc. (Plymouth Meeting, PA) and dissolved in DMSO at a stock concentration of 10 mM, aliquoted and stored at −80°C. Other reagents used in this study were obtained from the following sources: Proteasome inhibitor Vel (BD Biosciences, San Jose, CA); N-acetyl-L-cysteine (NAC), N-ethylmaleimide (NEM, Sigma-Aldrich Inc., St. Louis, MO); Proteasome-Glo^™^ Chymotrypsin-like Cell-Based Assay, Proteasome-Glo^™^ Trypsin-like Cell-Based Assay, Proteasome-Glo^™^ Caspase-like Cell-Based Assay (Promega Bioscience, Madison, WI); Caspase Inhibitor Z-VAD-FMK (BIOMOL International LP, Plymouth Meeting, PA); Suc-Leu-Leu-Val-Tyr-aminomethylcoumarin (Suc-LLVY-AMC), Z-Leu-Leu-Glu-AMC (Z-LLE-AMC), Boc-Leu-Arg-Arg-AMC (Boc-LRR-AMC), 20S and 26S human Proteasome, HA-Ubiquitin-Vinyl Sulfone (HA-Ub-VS), Tetra-ubiquitin (K48-linked), Ubiquitin-AMC (U550) (Boston Biochem, Cambridge, MA). Control siRNA-A, RPN11 siRNA (h), UCH-L5 siRNA (h), USP14 siRNA (h) (Santa Cruz Biotechnology, Santa Cruz, CA). Antibodies used in this study were purchased from following sources: anti-ub (P4D1), anti-GFP (B-2) (Santa Cruz Biotechnology, Santa Cruz, CA); anti-p21 Waf1/Cip1 (DCS60), anti-caspase3 (8G10), anti-caspase8 (1C12), anti-caspase 9 (C9), anti-PARP, anti-CHOP (L63F7), anti-histone H3 (D1H2) XP^™^, anti-K48-linkage specific polyubiquitin (D9D5), anti-K63-linkage specific polyubiquitin (D7A11), anti-NF-κB p65 (L8F6) (Cell Signaling Technology, Beverly, MA, USA); anti-RPN11, anti-UCHL5/Uch37 (Epitomics); anti-USP14 (C-term) (ABGENT); anti-GAPDH, anti-c-jun (N85), anti-HA-tag, anti-caspase12 (P99), anti-cleaved caspase-3, -8, -9 (Bioworld Technology, Inc.). MTS assay (CellTiter 96 Aqueous One Solution reagent) was purchased from Promega Corporation (Madison, WI, USA). PI and Annexin V-FITC apoptosis Detection Kit and cell apoptosis Rhodamine 123 Detection Kit were purchased from Keygen Company (Nanjing, China). DCFH-DA was purchased from Beyotime Institute of Biotechnology (Jiangsu, China). Enhanced chemiluminescence (ECL) reagents were purchased from Santa Cruz Biotechnology Inc. (Santa Cruz, CA). Lipofectamine^™^ RNAiMAX and Lipofectamine 2000 were purchased from Invitrogen Corporation.

### Western blot analysis

Whole cell lysates were prepared in RIPA buffer supplemented with 10 mM β-glycerophosphate, 1 mM sodium orthovanadate, 10 mM NaF, 1 mM phenylmethylsulfonyl fluoride (PMSF), and 1×Roche Complete Mini Protease Inhibitor Cocktail (Roche, Indianapolis, IN). To detect the level of IκBα in the cytosol and NF-κB p65 in the nuclear, cytosol and the nuclear fractions were extracted by using a kit from Nanjing Keygen (Nanjing, China). Western blotting was performed as we previously described [63], using specific primary antibodies as indicated and horseradish peroxidase (HRP)-conjugated appropriate secondary antibodies as indicated.

### Peptidase activity assay

About 4,000 cells were treated with Aur at various concentrations at 37°C for 6 h. The drug-treated cells were then incubated with the Glo Cell-Based Assay Reagent (Promega Bioscience, Madison, WI) for 10 minutes. Luminescence generated from each reaction was detected with microplate reader (Varioskan Flash 3001, Thermo, USA). *In vitro* proteasome peptidases were detected as previously reported [64]. Briefly, These were performed as we previously described. A 20 μL of Tris-HCl buffer (pH 7.4) containing purified 26S proteasome (0.5 nM) were added to a total volume of 180 μL Tris-HCl (pH 7.4) reaction buffer containing the synthetic fluorogenic peptides (Boston Biochem, Cambridge, MA). The reaction mixture was then incubated at 37°C for 90 min and analyzed for the fluorescence intensity of the free AMC using a luminescence microplate reader (Varioskan Flash 3001, Thermo, USA).

### Deubiquitinase activity assay

This was performed as reported [17]. Briefly, cell lysate (5 μg) or 26S proteasomes (25 nM) was solved in ice-cold DUB buffer containing 50 mmol/L Tris-HCl (pH 7.5), 250 mM sucrose, 5 mM MgCl_2_, and 1 mM PMSF and pretreated with Aur (2 μM) or 2 mM NEM for 15 minutes, then incubated with Ub-AMC substrate in a 100 μL reaction volume at 25°C. AMC release generated from the cleaved substrate was temporally recorded with microplate reader (Varioskan Flash 3001, Thermo, USA).

### Computational modeling

In order to understand the intermolecular interaction between Chloro (triethylphosphine) gold and deubiquitinase RPN11, a molecular docking study was performed with CDOCKER protocol of Discovery Studio 2.0 [65]. Taking into account the possible hydrolysis of the compound Chloro (triethylphosphine) gold (L1), two compounds Chloro (triethylphosphine) gold (L1) and (triethylphosphine) gold cation (L2) were selected as the docking ligands. The geometry structures of two compounds (L1 and L2) were respectively optimized using the density functional theory (DFT) calculations at the B3LYP/LANL2DZ level. The NPA charges were obtained by the natural orbital population analysis (NPA). These quantum chemistry calculations were performed by using the Gaussian 03 package of programs [66]. The conformations with the lowest energy were selected as initial docking ligand structures. During the whole docking process, the proteins UCHL5 (PDB ID: 3RIS) and USP14 (PDB ID: 2AYO) were rigid, while ligands L1 and L2 were flexible. The Ludi Energy Estimate was used for scoring the docked poses. The ligand-pose which corresponded to the highest Ludi score was selected as the most probable binding conformation [67]. All parameters used in calculation were default except for explained. As previous literatures [68, 69] show that the catalytic triad in the active site of UCHL5 is formed by Cys88, His164 and Asp179, while that of USP14 is formed by Cys113, His434 and Asp450, the Input Site Spheres were respectively centered on the two catalytic triads with radius 12Å.

### Ubiquitin chain disassembly

*In vitro* disassembly of purified tetra-ubiquitin chains (K48- or K63- linked) was performed as described earlier [17]. 26S proteasomes (25 nM) were pre-incubated with Aur (2, 40 μM) for 10 min *in vitro*. Then K48- or K63-linked Ub chains (1 μg) were added into the DUB buffer for 1 h at 37°C. The extent of chain disassembly was assessed by western blot analysis.

### Active DUB labeling assays

This was performed as previously reported [70, 71]. 26S proteasomes (25 nM) were treated with Aur (2, 40 μM) for 10 minutes before they were incubated with HA-UbVS for 1 h at 37°C, followed by boiling in reducing sample buffer and resolving by SDS-PAGE. After protein transfer to PVDF membranes, HA immunoblotting was used to detect HA-UbVS labeled DUBs.

### siRNA transfection

Three siRNAs against human RPN11, UCHL5 and USP14, constructed and ordered from Guangzhou Ribobio Co. Ltd, were used to transfect HepG2 and MCF-7 cells. For each transfection sample, oligomer-Lipofectamine^™^ 2000 complexes were prepared. The oligomer-Lipofectamine^™^ 2000 complexes were added to each well containing cells and medium. Medium was changed after 6 h, and the cells were incubated at 37°C in a CO_2_ incubator for 24 h or 48 h, followed by Aur treatment as indicated. Cells were collected for Western blot assay as described above.

### Cell death assay

Apoptosis was determined by flow cytometry using Annexin V-fluoroisothiocyanate (FITC)/PI double staining [64]. Cells were treated, then collected and washed with binding buffer, then incubated in working solution (100 μl binding buffer with 1.0 μl Annexin V-FITC) for 15 min in dark. PI was added just before flow cytometric analysis. The double stained cells were also imaged with an inverted fluorescence microscope equipped with a digital camera (Axio Obsever Z1, Zeiss, Germany).

### Cell viability assay

MTS assay (CellTiter 96Aqueous One Solution reagent; Promega, Shanghai, China) was used to test cell viability according to previously reported [64]. Briefly, 2×10^5^/ml cells in 100 μl were treated with Aur for 24 or 48 h. 4 h before culture termination, 20 μl MTS was added to the wells. The absorbance density was read on a 96-well plate reader at wavelength 490 nm. IC_50_ values were calculated.

### Measurement of mitochondrial membrane integrity

The mitochondrial membrane potential of Aur-treated and untreated cells was assayed by using rhodamine-123 staining as we previously reported [72]. Cells were treated with Aur for 12 h and stained with 1 μM of rhodamine-123 for 1 h at 37°C. Following the staining, the cells were washed with PBS twice, and then harvested for flow cytometry analysis.

### *In vitro* complex formation of Aur with NAC and HPLC analysis

A 1 mM solution of Aur was mixed with a 10 mM solution of NAC and in a PBS(phosphate buffer saline, pH7.4). Prepared mixtures were incubated for 48 h in room temperature. Incubation mixtures were collected and then ﬁltered through a 0.45μm Advantec ﬁlter and a 20 μl volume was injected into the HPLC system. Chromatographic analysis was performed with a Shimadzu LC-10A liquid chromatograph, SPD-10A variable wavelength diode-array detector, SCL-10A system controller, SIL-10A automatic sample injector and a dual-pump LC-10AT binary system. Data was collected digitally with Shimadzu LCsolution software. The analysis was carried out on an ODS column (Shim-pack, 5μm, 4.6×250 mm I.D, Shimadzu, Japan). The mobile phase consisted of a mixture of acetonitrile-0.1% phosphoric acid (60:40 v/v %), and the column temperature was maintained at 25°C. A constant mobile phase with a flow-rate of 1.0 ml/min was employed throughout the analyses. The ultraviolet (UV) detector was set at 254 nm.

### Nude mouse xenograft model

Nude Balb/c mice were bred at the animal facility of Guangzhou Medical University. The mice were housed in barrier facilities with a 12 h light dark cycle, with food and water available ad libitum. 3×10^7^ of HepG2 or MCF-7 cells was inoculated subcutaneously on the flanks of 5-week-old male nude mice. After 72 h of inoculation, mice were treated with either vehicle (10% DMSO, 30% Cremophor ELand 60% NaCl) or Aur (6 mg/kg/day) for totally 15 or 21 days, respectively. Tumors were measured every other day with use of calipers. Tumor volumes were calculated as previously reported [66]. Aur was dissolved in the buffer with 10% DMSO, 30% Cremophor EL and 60% NaCl. All animal studies were conducted with the approval of the Institutional Animal Care and Use Committee of Guangzhou medical University.

### Immunohistochemical staining

Formalin-fixed xenografts were embedded in paraffin and sectioned according to standard techniques as we previously reported [72]. Tumor xenograft sections (4 μm) were immunostained using the MaxVision kit (Maixin Biol) according to the manufacturer's instructions. The primary antibodies were against ubiquitin, K-48- or K63-linked polyubiquitin, p21 and c-Jun. 50 μl MaxVisionTM reagent was applied to each slide. Color was developed with 0.05% diaminobenzidine and 0.03% H_2_O_2_ in 50 mM Tris-HCl (pH 7.6), and the slides were counterstained with hematoxylin. A negative control for every antibody was also included for each xenograft specimen by substituting the primary antibody with preimmune rabbit serum.

### Cell culture and sample collection

Peripheral blood samples of normal controls were obtained from Guangzhou Blood Center and peripheral bone marrow samples of AML patients were obtained from discarded material utilized for routine laboratory tests at the Department of Hematology, Guangzhou First Municipal People's Hospital of Guangzhou Medical University; The use of these materials is approved by the Institutions with the permission of the patients and volunteers. Totally six patients with AML and six volunteers were recruited in this preclinical study. Mononuclear cells were isolated by Ficoll-Paque (Pharmacia, Uppsala, Sweden) density gradient. Mononuclear cell fraction was cultured in RPMI 1640 culture medium with 15% FBS.

### ROS measurement

ROS production was detected as previously reported [73]. HepG2 and MCF-7 cells were treated with Aur (0.5 μM) and/or NAC (5 mM) for 12 h. The cells were harvested and incubated with the free serum medium with addition of 10 μM of DCFH-DA for 20 min at 37°C in the dark. In the presence of ROS, DCFH penetrates the cells and is in turn oxidized to DCF. DCF fluorescence was detected by flow cytometry.

### Statistical analysis

All experiments were performed at least thrice, and the results were expressed as Mean±SD where applicable. GraphPad Prism 4.0 software (GraphPad Software) was used for statistical analysis. Comparison of multiple groups was made with one-way ANOVA followed by Tukey's test or Newman-Kueuls test. *P* value of <0.05 was considered statistically significant.

## SUPPLEMENTARY MATERIAL AND FIGURES


