# Supplementary material for: Clinically used antirheumatic agent auranofin is a proteasomal deubiquitinase inhibitor and inhibits tumor growth
Source: Oncotarget. 2014 Jun 18;5(14):5453–71. doi: 10.18632/oncotarget.2113 (PMC4170648; doi:10.18632/oncotarget.2113)
Supplement: Supplementary file 1 [file oncotarget-05-5453-s001.pdf]

Clinically used antirheumatic agent auranofin is a proteasomal deubiquitinase inhibitor and inhibits tumor growth

Supplementary Material

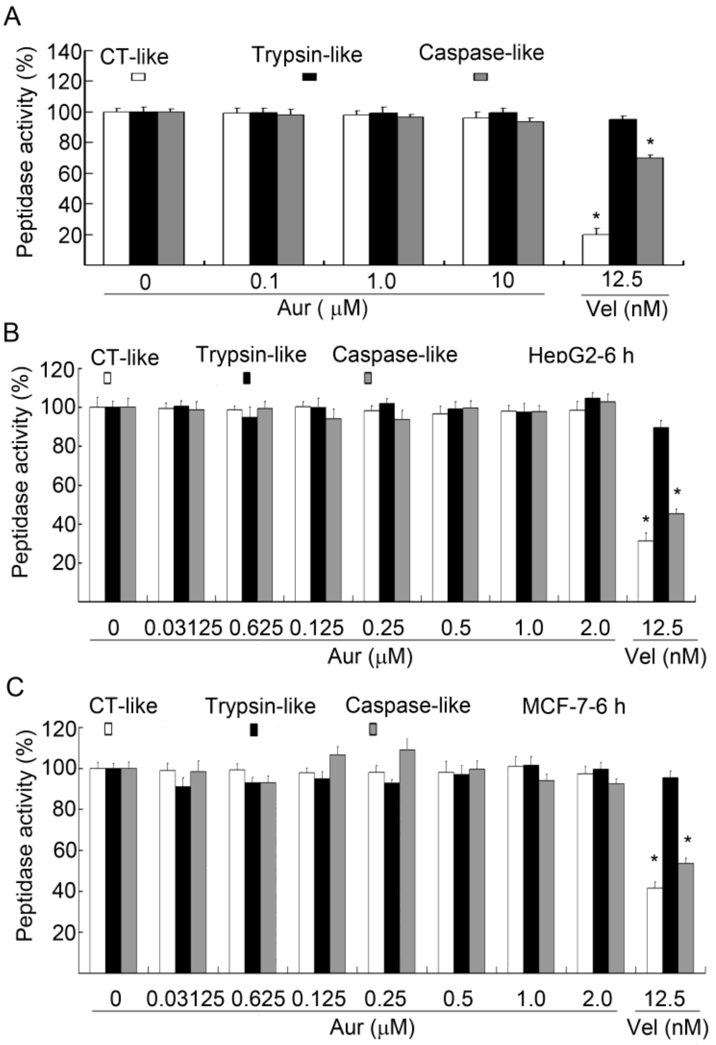

Figure S1: Auranofin (Aur) does not inhibit proteasome peptidase activities both *in vitro* and *in situ*. (A) Purified 20S and 26S proteasomes were treated with auranofin at the indicated doses in a Tris reaction system (pH 7.4). The chymotrypsin-like (CT-like) peptidase activity was measured using specific synthetic fluorogenic substrates. Mean±SD (n=3). **(B, C)** Effect of auranofin on the three proteasome peptidase activities in live cells. HepG2 and MCF-7 cells were incubated with indicated doses of Aur or velcade (12.5 nM) for 6 h, peptidase (CT-like, trypsin-like and caspase-like) activities were detected in live cells. Relative peptidase activities were shown. Mean±SD (n=3). *P<0.05, *versus* control.

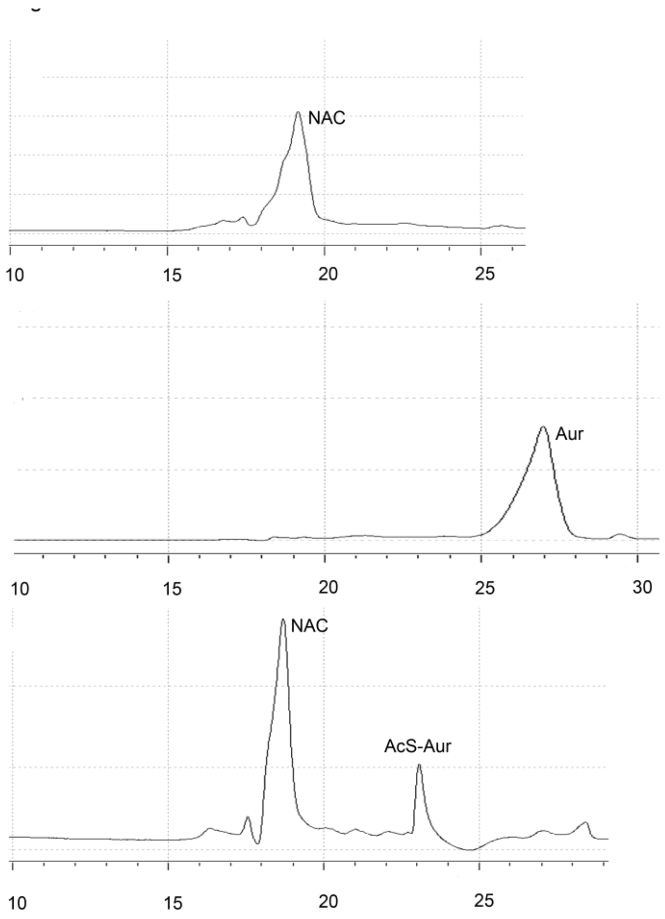

Figure S2: Representative HPLC chromatography. Chemical compounds were detected by HPLC and representative chromatograph images were shown. NAC standard (upper); auranofin standard (middle); products of chemical reaction with NAC and auranofin for 48 h (lower).

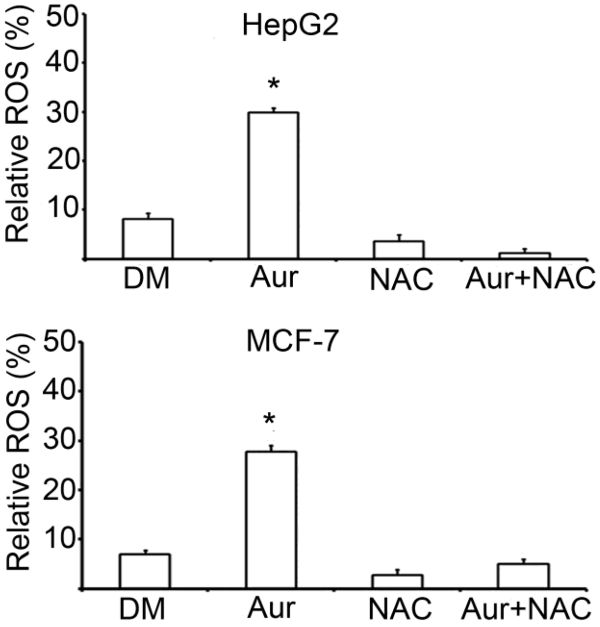

Figure S3: NAC blocked Aur-mediated ROS generation. HepG2 and MCF-7 cells were treated with Aur (0.5 μM) and /or NAC (5 mM) for 12 h. ROS was detected by flow cytometry. Relative level of ROS was shown. Mean±SD (n=3). *$P<0.05$, compared with other treatments.
